# *Staphylococcus saccharolyticus*: An Overlooked Human Skin Colonizer

**DOI:** 10.3390/microorganisms8081105

**Published:** 2020-07-23

**Authors:** Charlotte M. Ahle, Kristian Stødkilde, Mastaneh Afshar, Anja Poehlein, Lesley A. Ogilvie, Bo Söderquist, Jennifer Hüpeden, Holger Brüggemann

**Affiliations:** 1 Beiersdorf AG, Research & Development, Front End Innovation, 20245 Hamburg, Germany; charlotte.ahle@beiersdorf.com (C.M.A.); jennifer.huepeden@beiersdorf.com (J.H.); 2 Department of Microbiology and Biotechnology, University of Hamburg, 22609 Hamburg, Germany; 3 Department of Biomedicine, Aarhus University, 8000 Aarhus, Denmark; kst@biomed.au.dk (K.S.); m.afshar@biomed.au.dk (M.A.); 4 Department of Genomic and Applied Microbiology, Institute of Microbiology and Genetics, University of Göttingen, 37073 Göttingen, Germany; anja.poehlein@biologie.uni-goettingen.de; 5 Max Planck Institute for Molecular Genetics, 14195 Berlin, Germany; logilvie@molgen.mpg.de; 6 Department of Laboratory Medicine, Clinical Microbiology, Faculty of Medicine and Health, Örebro University, S-701 82 Örebro, Sweden; bo.soderquist@oru.se

**Keywords:** *Staphylococcus saccharolyticus*, coagulase-negative staphylococci, skin microbiota, skin microbiome, amplicon next generation sequencing

## Abstract

Coagulase-negative staphylococcal species constitute an important part of the human skin microbiota. In particular, facultative anaerobic species such as *Staphylococcus epidermidis* and *Staphylococcus capitis* can be found on the skin of virtually every human being. Here, we applied a culture-independent amplicon sequencing approach to identify staphylococcal species on the skin of healthy human individuals. While *S. epidermidis* and *S. capitis* were found as primary residents of back skin, surprisingly, the third most abundant member was *Staphylococcus saccharolyticus*, a relatively unstudied species. A search of skin metagenomic datasets detected sequences identical to the genome of *S. saccharolyticus* in diverse skin sites, including the back, forehead, and elbow pit. Although described as a slow-growing anaerobic species, a re-evaluation of its growth behavior showed that *S. saccharolyticus* can grow under oxic conditions, and, in particular, in a CO_2_-rich atmosphere. We argue here that *S. saccharolyticus* was largely overlooked in previous culture-dependent and -independent studies, due to its requirement for fastidious growth conditions and the lack of reference genome sequences, respectively. Future studies are needed to unravel the microbiology and host-interacting properties of *S. saccharolyticus* and its role as a prevalent skin colonizer.

## 1. Introduction

In recent years, new discoveries regarding the composition and functionality of the human skin microbiota have been made, that have enabled a more comprehensive description of this ecosystem [1,2,3]. Studies highlighted the diversity and uniqueness of the collection of skin microorganisms with essential roles in protection against harmful pathogens, maintaining skin homeostasis, and priming our immune system [3,4,5,6].

Coagulase-negative staphylococci (CoNS) constitute an important part of the human skin microbiota. Culture-dependent and -independent studies have highlighted the ubiquity of CoNS, which colonize mostly moist and sebaceous areas of the skin. In this regard, the CoNS species *Staphylococcus epidermidis, Staphylococcus capitis,* and *Staphylococcus hominis* occupy many human skin sites [1,2,6,7,8,9]. Other CoNS species, such as *Staphylococcus haemolyticus, Staphylococcus lugdunensis,* and *Staphylococcus warneri,* can be found in lower amounts, varying from person to person and from skin site to skin site [1,2,9,10]. Some other CoNS species, such as *Staphylococcus equorum,* are primarily found in food products [11], but are also transient colonizers of human skin. 

Culture-dependent approaches have been often applied in the past to isolate major skin residents. Such approaches are often biased, as cultivation results do not reflect the true distribution of the individual members of the microbiota [12,13]. The bias is introduced due to the chosen growth media, as well as the conditions of growth, such as O_2_ and CO_2_ concentrations, growth temperature, and cultivation time. Fast-growing microorganisms have a growth advantage, and directly or indirectly inhibit the growth of slow-growing microorganisms [6]. Therefore, culture-independent studies employing next generation sequencing (NGS)-based approaches are more frequently used in recent years. Using 16S rRNA gene amplicon-NGS, it was shown that the genus *Staphylococcus* is the third most abundant genus on the skin [14]. In addition, shotgun NGS studies further unraveled the diversity and individuality of staphylococcal species on the skin, and also provided insights into the strain level distribution of these CoNS species [1,2]. Such studies also highlighted the existence of microbial dark matter in the form of unidentified bacterial skin residents. For instance, the study of Oh et al. [1] has identified several uncharacterized genomes (assembled from shotgun NGS data) of unknown species, possibly belonging to the genera *Corynebacterium, Cutibacterium,* and *Staphylococcus*. Thus, it can be expected that species exist on the skin that cannot be easily cultivated by standard conditions.

In this context, we have recently described the genomes of seven strains of *Staphylococcus saccharolyticus*, an unusual CoNS species, regarding its growth properties [15]. Unlike almost all other CoNS species known to date, *S. saccharolyticus* largely depends on anaerobic conditions for growth, and requires fastidious growth media and prolonged cultivation time (>3 days). To date, this species has been relatively uncharacterized, with limited reports on its association with implant-associated infections [15] and bacteremia [16]. Interestingly, however, culture-dependent studies have suggested that this species may also be a resident of the skin microbiota [17,18].

Here, we further investigated the composition and relative abundance of staphylococcal species on human skin. For this, we applied an amplicon-NGS approach based on a *Staphylococcus*-specific gene fragment, to survey its presence on human back skin samples from healthy volunteers. We detected an unprecedented high relative abundance of *S. saccharolyticus* in these samples. Supported by investigation of existing skin-derived metagenomic datasets, we posit that *S. saccharolyticus* constitutes a common member of the human skin microbiota.

## 2. Materials and Methods 

### 2.1. Study Design and Sampling 

Skin swab samples from 19 volunteers (female, *n* = 11; male, *n* = 8) with an age range of 22–43 years were taken from the upper back. None of the volunteers had a history of skin disease; none had undergone treatment with topical medicine or antibiotics during the last six months. Written informed consent was obtained from all volunteers, and the study was approved by International Medical & Dental Ethics Commission GmbH (IMDEC).

An area of 25 cm^2^ on the upper back was sampled with a cotton swab pre-moistened in aqueous sampling buffer containing disodium phosphate (12.49 g/L, Merck, Darmstadt, Germany), potassium dihydrogen phosphate (0.63 g/L, Merck, Darmstadt, Germany), and Triton X-100 (1 g/L, Merck, Darmstadt, Germany). After sampling, the swab was transferred into a sterile tube containing 2 mL of sampling buffer; the swab was vigorously shaken in the sampling buffer and then removed. Skin swab material was stored at -20 °C until further processing. 

### 2.2. DNA Extraction

DNA from the 2 mL sample was extracted using the DNeasy PowerSoil Kit (QIAGEN, Hilden, Germany) following the manufacturer’s protocol, with an additional cell lysis step using lysostaphin (0.05 mg/mL, Merck, Darmstadt, Germany) and lysozyme (9.5 mg/mL, Merck, Darmstadt, Germany) prior to extraction. DNA concentrations were measured using the Qubit dsDNA HS Assay (ThermoFisher Scientific, Waltham, MA, USA) with a Qubit fluorometer following the manufacturer’s instructions. 

### 2.3. Amplicon PCR 

A fragment of the *tuf* gene present in the genomes of all staphylococcal species available in GenBank (as of December 2019) was used for species identification, analogous to a previous study using a different *tuf* gene fragment [19]. The target sequence was amplified using *tuf*-specific primers that contained MiSeq adapter sequences: tuf2_F, 5′-TCGTCGGCAGCGTCAGATGTGTATAAGAGACAGACAGGCCGTGTTGAACGTG-3′; tuf2_R, 5′-GTCTCGTGGGCTCGGAGATGTGTATAAGAGACAGACAGTACGTCCACCTTCACG-3′.

PCR reaction mixtures were made in a total volume of 25 µl and comprised 5 µl of DNA sample, 2.5 µl AccuPrime PCR Buffer II (Invitrogen, Waltham, MA, USA), 1.5 µl of each primer (10 µM) (DNA Technology, Risskov, Denmark), 0.15 µl AccuPrime Taq DNA Polymerase High Fidelity (Invitrogen, Waltham, MA, USA), and 14.35 µl of PCR grade water. The PCR reaction was performed using the following cycle conditions: an initial denaturation at 94 °C for 2 min, followed by 35 cycles of denaturation at 94 °C for 20 sec, annealing at 55 °C for 30 sec, elongation at 68 °C for 1 min, and a final elongation step at 72 °C for 5 min. PCR products were verified on an agarose gel and purified using the Qiagen Generead^TM^ Size Selection kit (Qiagen, Hilden, Germany). The concentration of the purified PCR products was measured with a NanoDrop 2000 spectrophotometer (ThermoFisher Scientific, Waltham, MA, USA). 

### 2.4. Amplicon Next Generation Sequencing

PCR products were used to attach indices and Illumina sequencing adapters using the Nextera XT Index kit (Illumina, San Diego, CA, USA). Index PCR was performed using 5 µl of template PCR product, 2.5 µl of each index primer, 12.5 µl of 2x KAPA HiFi HotStart ReadyMix, and 2.5 µl PCR grade water. The thermal cycling scheme was as follows: 95 °C for 3 min, 8 cycles of 30 sec at 95 °C, 30 sec at 55 °C, and 30 sec at 72 °C, and a final extension at 72 °C for 5 min. Quantification of the products was performed using the Quant-iT dsDNA HS assay kit (ThermoFisher Scientific, Waltham, MA, USA) and a Qubit fluorometer, following the manufacturer’s instructions. MagSi-NGS^PREP^ Plus Magnetic beads (Steinbrenner Laborsysteme GmbH, Wiesenbach, Germany) were used for purification of the indexed products as recommended by the manufacturer, and normalization was performed using the Janus Automated Workstation from Perkin Elmer (Perkin Elmer, Waltham, MA, USA). Sequencing was conducted using an Illumina MiSeq platform with dual indexing and the MiSeq reagent kit v3 (600 cycles), as recommended by the manufacturer. 

### 2.5. Bioinformatics

FASTQ sequences obtained after demultiplexing the reads and trimming the primers were imported into QIIME2 (v. 2019.7) [20]. Sequences with an average quality score lower than 20 or containing unresolved nucleotides were removed from the dataset with the split_libraries_fastq.py script from QIIME. The paired-end reads were denoised and chimeras removed with DADA2 via QIIME2, and a feature table was generated [21]. These features were then clustered with VSEARCH at a threshold of 99% identity against an in-house generated *tuf* allele database that contained all *tuf* alleles from all staphylococcal genomes available in GenBank (as of December 2019). Data were normalized, and figures were prepared in R with the packages ggplot2 and gplots.

### 2.6. Metagenome Database Search Strategy

The presence of sequences similar to *S. saccharolyticus* within available metagenomes deposited in the Sequence Read Archive (SRA) was initially assessed using the online tool at www.searchsra.org to provide a broad overview of datasets with matches. A more detailed investigation of the level of representation of *S. saccharolyticus* in existing human skin and other human-associated metagenomic datasets (identified within the initial search of the SRA) was then conducted by mapping pooled sequencing reads from metagenomic datasets against the *S. saccharolyticus* genome sequence (strain 05B0362; GenBank accession number: QHKH00000000). Sequencing reads were obtained from the SRA and processed using Geneious Prime 2020 to remove low quality reads (Trim using BBDuk; min. 50 bp) and duplicates (Dedupe from BBTools), with default parameters. The resulting collections of high-quality reads were mapped against the genome sequences of *S. saccharolyticus* 05B0362, *Cutibacterium acnes* strain ATCC 6919 (NZ_CP023676.1) and *Staphylococcus epidermidis* strain ATCC 14990 (NZ_CP035288.1) using the Geneious Prime 2020 map to reference tool with the following criteria: 100% identity; no gaps or mismatches; maximum ambiguity = 1. For each metagenomic dataset mapped, the total number of reads mapped to each reference genome was normalized by the total size of the dataset, to provide reads mapped per megabase DNA. 

A more targeted search strategy was also applied using an *S. saccharolyticus*-specific gene as a query to search metagenomic datasets derived from back skin [1]. The *hya* gene encoding a hyaluronate lysase was chosen (locus tag DMB78_01130 in the genome of strain 05B0362), due to its low average nucleotide identify with *hya* genes from other staphylococcal species. The search was performed as an SRA nucleotide BLAST. The gene was considered as being present when the coverage exceeded 40%.

### 2.7. S. Saccharolyticus Growth

*S. saccharolyticus* (strain DVP5-16-4677) was grown on Fastidious anaerobic agar (FAA) plates (LAB M, Bury, UK) and incubated anaerobically at 37 °C for 4 days. For liquid growth, brain-heart infusion-yeast broth supplemented with 0.05% (w/vol) cysteine (BHCY broth) was used. The following growth conditions were evaluated and performed at 37 °C: anoxic conditions (Oxoid AnaeroGen System; ThermoFisher Scientific, Waltham, MA, USA), and oxic conditions with and without CO_2_-supplementation (Oxoid CO_2_ Gen system; ThermoFisher Scientific, Waltham, MA, USA). Optical density (OD) data at 600 nm was determined until the stationary growth phase. 

## 3. Results

### 3.1. Amplicon Sequencing of a Tuf Gene Fragment Identified Staphylococcal Species Diversity on Back Skin Samples

**Figure 1:**
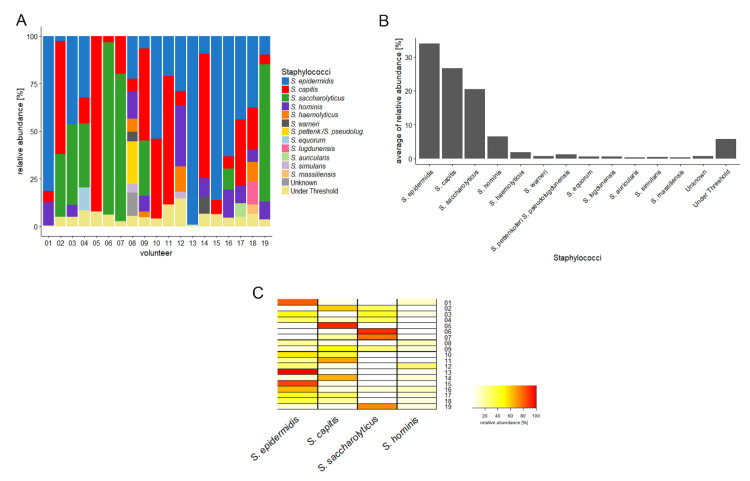
Diversity and abundance of staphylococcal species in back skin samples, based on amplicon next generation sequencing (NGS) data. (**A**) Relative abundance of staphylococcal species for each volunteer (*n* = 19). Twelve staphylococcal species were identified in the cohort using the tuf amplicon-NGS approach. The four most abundant species in the cohort were *Staphylococcus epidermidis* (in blue), *Staphylococcus capitis* (in red), *Staphylococcus saccharolyticus* (in green) and *Staphylococcus hominis* (in purple). (**B**) The average relative abundance of the identified 12 staphylococcal species is shown; *S. epidermidis* was detected with an average abundance of 34.0%, *S. capitis* with 26.6%, *S. saccharolyticus* with 20.5%, and *S. hominis* with 6.5%. (**C**) The relative abundance of the four most prevalent staphylococcal species is shown for each back skin sample in a heat map. *S. epidermidis* was detected in four samples with a very high abundance (>60% of all reads); *S. capitis* and *S. saccharolyticus* were detected with such a high abundance in three samples each.

Amplicon sequencing based on a *tuf* gene fragment was applied on swab material derived from the upper back of 19 healthy volunteers, to determine the diversity and relative abundance of staphylococcal species. In total, twelve different staphylococcal species were identified (Figure 1A and Table S1). The majority of samples contained two or more staphylococcal species, with single species found in only two samples (*S. capitis* and *S. epidermidis,* respectively). The four most abundant staphylococcal species identified were *S. epidermidis* (average abundance 34.0%), *S. capitis* (26.6%), *S. saccharolyticus* (20.5%), and *S. hominis* (6.5%) (Figure 1B). *S. saccharolyticus* was identified in 8 out of the 19 samples (42%) tested and, if present, was a dominant species, comprising a minimum of 10.9% and a maximum of 90.4% of the total reads (Figure 1C).

### 3.2. Presence of *S. Saccharolyticus* in Previous Metagenome Studies

**Figure 2:**
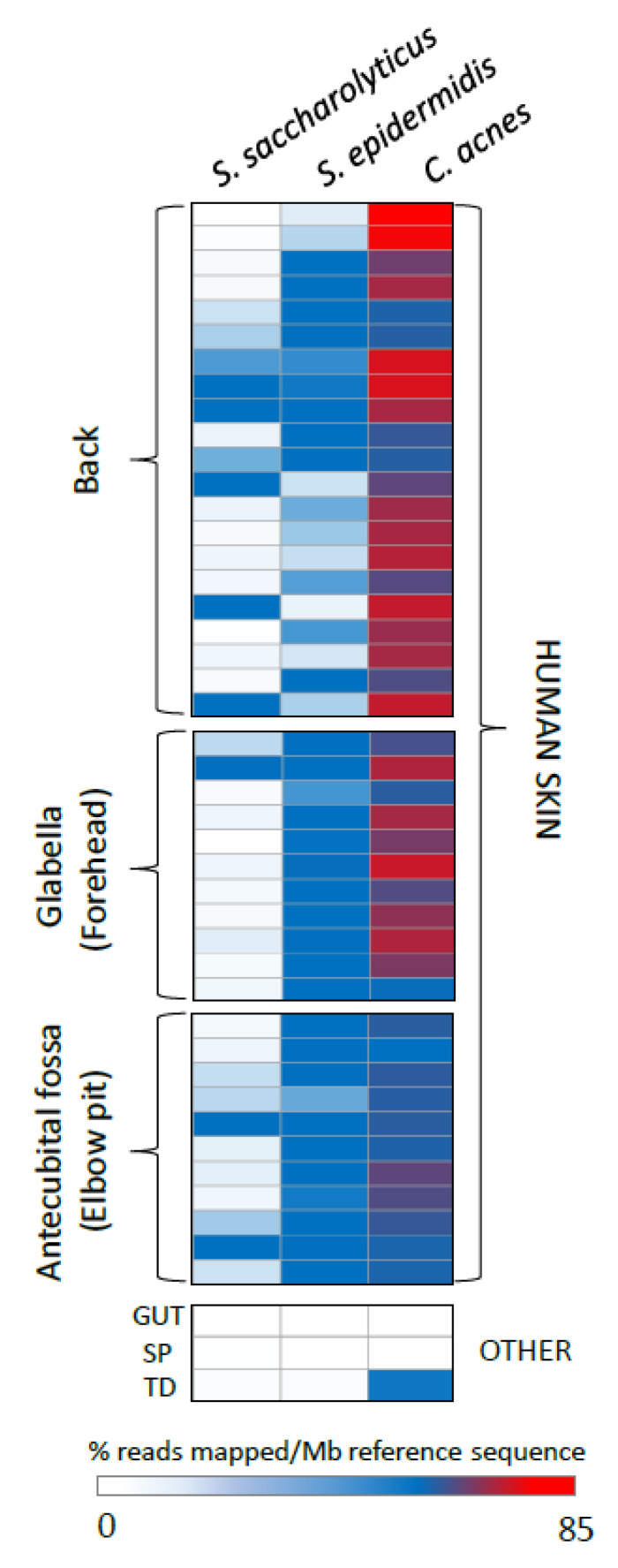
Representation of *S. saccharolyticus* in human skin and human-associated metagenomes. Heat map showing relative representation of S. *saccharolyticus* in metagenomes from human skin and other human associated environments. Reads from each reference metagenome were mapped to the genome sequences of *S. saccharolyticus, S. epidermidis,* and *Cutibacterium acnes* using high stringency criteria (100% identity; no gaps; max. ambiguity 1). The number of reads mapped was normalized for size of reference datasets (expressed as % of reads mapped/Mb reference sequence). OTHER: non-human skin metagenomes; GUT: human gut; SP: supragingival plaque; TD: tongue dorsum. Details of the metagenomes utilized are provided in Table S3.

Given that *S. saccharolyticus* was detected in 8 out of 19 back skin samples tested, we next decided to determine the prevalence and distribution of sequences with similarity to *S. saccharolyticus* within existing skin metagenomic datasets, using two different search strategies. First, sequencing reads from all skin metagenomes derived from back, forehead, and armpit samples (*n* = 43) [1,2], available within the Sequence Read Archive (SRA), were mapped to the *S. saccharolyticus* genome with high stringency (100% identity; no gaps or mismatches; maximum ambiguity = 1). As a comparison, we also mapped the same reads to the genomes of two bacterial species known to be abundant on human skin, *Cutibacterium acnes* and *S. epidermidis.* Reads mapping to the *S. saccharolyticus* genome were found in all 43 skin metagenomic datasets searched (Figure 2). The percentage of reads mapping to *C. acnes* was significantly higher for all datasets searched, as compared to *S. saccharolyticus* and *S. epidermidis*. To compare relative abundance profiles, we also conducted a survey of a limited set of other human-associated metagenomic datasets (originating from the human gut, tongue dorsum, and supragingival plaque) identified as carrying sequences with similarity to *S. saccharolyticus,* using the SRA metagenomic search tool. Sequencing reads from these human-associated metagenomic datasets were also found to map to all bacterial genomes assessed, but at magnitudes of order lower levels than observed for human skin datasets. 

To complement the read mapping analyses, we also conducted a more targeted search of the shotgun NGS data derived from back skin samples of Oh el al., 2014 [1] and Oh et al., 2016 [2] (12 volunteers; samples taken at three time points per person), using the hyaluronate lyase gene of *S. saccharolyticus*. Using this approach, we found high-stringency matches (reads with 100% identity) within three of the twelve volunteers at multiple time points (Table S2).

### 3.3. Re-Evaluation of Growth Conditions for *S. Saccharolyticus*

**Figure 3:**
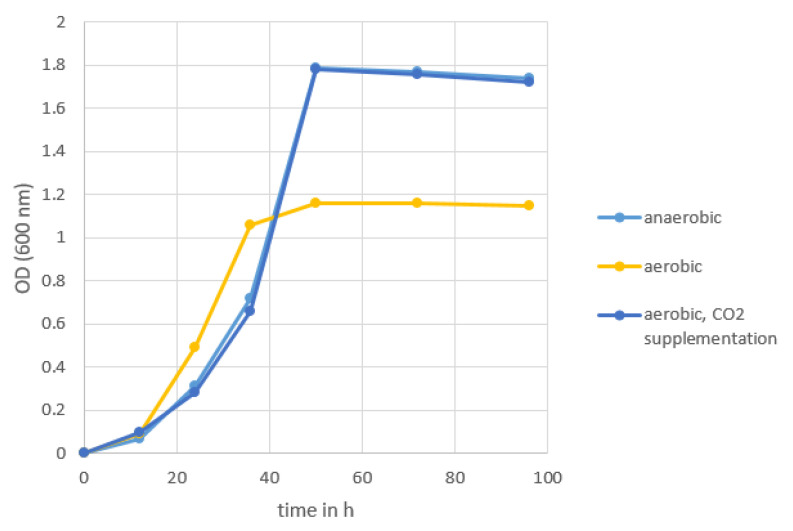
Growth of *S. saccharolyticus* in BHCY medium under different conditions. Light blue, anaerobic atmosphere (Oxoid-AnaeroGen system); dark blue, CO_2_-rich atmosphere (Oxoid-CO_2_ Gen system; generating ca. 6% CO_2_, ca. 15% O_2_); yellow, aerobic atmosphere. The experiment was replicated twice.

Our data indicate that *S. saccharolyticus* is widespread on human skin, can be detected within diverse skin sites, and may even be more abundant than *S. epidermidis* in some people. However, only a few studies have previously reported the presence of *S. saccharolyticus* on human skin. One possible reason for the previous lack of recognition is the difficulty of growing *S. saccharolyticus* under standard conditions. The organism cannot be detected on blood agar when incubated under aerobic conditions for 24 to 96 h (data not shown). Instead, it grows on Trypticase soy yeast (TSY) agar plates supplemented with 0.5% Tween-80 or FAA plates when incubated for 72 to 96 h under anaerobic conditions (data not shown). Not much is known about its growth in broth. Thus, we recorded the growth of *S. saccharolyticus* in brain-heart infusion-yeast broth supplemented with 0.05% cysteine (BHCY medium). Different incubation conditions were applied, including anaerobic and aerobic conditions and the supplementation with CO_2_ (Figure 3). Results showed that the organism grew almost equally as well under CO_2_-enriched conditions (approx. 6% CO_2_ and 15% O_2_), compared to anaerobic conditions. Growth yields were reduced under atmospheric conditions. 

## 4. Discussion

The skin microbiome affects the health-state of our skin. Understanding the bacterial composition on non-diseased skin is therefore of importance. Here, we focused on the staphylococcal composition of the human back skin. Similar to most previous studies [1,2], *S. epidermidis* was found to be the most abundant staphylococcal species on human back skin, followed by *S. capitis*. Surprisingly, *S. saccharolyticus* ranked third. This species has previously not been reported to be abundant on human skin. However, two studies from 1978 reported the presence of *S. saccharolyticus*, formerly named *Peptococcus saccharolyticus,* in forehead and armpit skin samples [17,18]. In these studies, around 20% of samples were found to be positive for *S. saccharolyticus*. The organism grew on TSY (supplemented with 0.5% Tween-80) agar plates after 4–7 days of incubation, with preference for anaerobic conditions. Identification (and differentiation from other CoNS) was based on cell and colony morphology, anaerobic growth preference, and a weak catalase activity. Biochemically, *S. saccharolyticus* cannot produce lactic acid from glucose (in contrast to other staphylococci); it can ferment glucose, fructose, and glycerol, but not maltose. Interestingly, Evans et al. [17] stated that it was puzzling that the organism was not recognized in past studies “in view of its prevalence”. The authors also suggested that the reason that previous skin studies have overlooked this organism was due to (i) the choice of the culture media, (ii) the need for prolonged incubation time, (iii) the preference for anaerobic culture conditions, and (iv) misidentification. Indeed, 40 years later, not much has changed in this regard. Most culture-dependent skin microbiota studies do overlook this microorganism, possibly due to inappropriate growth and cultivation conditions, as outlined by Evans et al. [17,18]. In addition, fast-growing species such as *S. epidermidis* might outcompete *S. saccharolyticus* on (standard) agar plates. This could explain why *S. saccharolyticus* was overlooked in culture-dependent studies. 

However, this does not explain why *S. saccharolyticus* was previously not detected in culture-independent studies, which are nowadays more frequently conducted. Many culture-independent studies are carried out using 16S rRNA gene amplicon sequencing, which relies on sufficient differences in the 16S rRNA gene to distinguish species. However, the 16S rRNA gene of *S. saccharolyticus* does not carry many single nucleotide polymorphisms (SNPs) that can easily distinguish it from other CoNS, namely *S. capitis* [15]. Thus, depending on the 16S rRNA gene amplification strategy (amplifying the V1, V2, V3, V4, V5, and the V6 region, respectively, or a combination thereof), *S. saccharolyticus* can be indistinguishable from *S. capitis*.

In recent years, shotgun sequencing was employed to identify the skin metagenome [1,2]. However, meaningful analyses of shotgun sequencing data rely on reference genomes of all skin microorganisms. Regarding *S. saccharolyticus*, no such reference genome was available before March 2019. Adding to the confusion, three genomes assigned to *S. saccharolyticus* were publicly available in GenBank before 2019, but these were wrongly classified as *S. saccharolyticus,* and actually belong to *S. capitis,* as previously noted [15]. They were recently correctly reassigned to *S. capitis*. Besides the genomes of seven *S. saccharolyticus* strains that have been sequenced during our previous study [15], the type strain of *S. saccharolyticus* (ATCC 14953/NCTC 11807) has been sequenced by two independent teams (WGS projects UHDZ01 and RXWW01), resulting in nearly identical genome sequences. Taken together, before March 2019 there was no correct reference genome sequence of *S. saccharolyticus* available; this has been now resolved. Thus, current and future shotgun sequencing studies should be able to identify *S. saccharolyticus* correctly. 

Here, we employed an amplicon sequencing method that is a modification from an existing method by Strube et al. [19]. The method is based on the amplification of a *tuf* gene fragment, with primers that were designed by Martineau et al. [22]. The *tuf* gene, encoding the elongation factor Tu, is a highly conserved gene in all staphylococcal species. We modified this method by choosing different amplification primers, since we noticed that the reverse primer designed by Martineau et al. [22] has two mismatches with the corresponding *tuf* gene sequence in the genome of *S. saccharolyticus*. It is thus likely that the primers of Martineau et al. [22] do not amplify the *tuf* gene of *S. saccharolyticus,* or only with reduced efficiency. 

Previous studies reported that *S. saccharolyticus* has a preference for anaerobic growth conditions [17,18]. Here, we showed that the bacterium can also grow under atmospheric conditions in broth, but with a reduced growth yield compared to anaerobic conditions. However, the growth in a CO_2_-rich atmosphere is comparable to the growth under anaerobic conditions. A mechanistic explanation of the effect of CO_2_ on bacterial growth was recently published by Fan et al. [23]. The authors investigated the growth-promoting effect of CO_2_ and the CO_2_-dependency of small colony variants of *S. aureus*, whose growth defect can be compensated by increased CO_2_/bicarbonate supplementation. They found that staphylococci employ a CO_2_-concentrating mechanism that enables them to grow at atmospheric CO_2_ levels. More specifically, they found that the system MpsAB is crucial for *S. aureus* growth at atmospheric CO_2_ levels. From a set of carefully designed experiments, they concluded that the MpsAB system represents a dissolved inorganic carbon transporter, or bicarbonate concentrating system, which creates an elevated concentration of intracellular bicarbonate. Consequently, a staphylococcal species without a functional MpsAB system would only grow poorly at atmospheric CO_2_ levels, and would rely on increased CO_2_ concentrations for accelerated growth. As we previously noted that the genome of *S. saccharolyticus* contains over 300 frameshift mutations [15], we checked the *mpsAB* genes. Indeed, the *mpsAB* genes in the genome of *S. saccharolyticus* are frameshifted, and thus the MpsAB system is most likely non-functional in *S. saccharolyticus*. In conclusion, the lack of a functional CO_2_-concentrating system in *S. saccharolyticus* is a likely explanation for its insufficient growth under atmospheric conditions. This can be compensated by providing increased CO_2_ concentrations.

Many questions remain, e.g., regarding the preferred niche of S. saccharolyticus on human skin and its evolutionary history that can be regarded as an example of reductive evolution, indicative of the massive genome decay [15]. Genomic modifications such as genome decay are often a result of a relative recent lifestyle change, e.g., the adaptation to a new host or a new niche within a host, associated with a strict(er) host dependency [24,25,26]. What could a scenario for the evolutionary history of *S. saccharolyticus* look like? In this regard, an interesting feature of *S. saccharolyticus* is the presence of a hyaluronate lyase (Hya), which is absent in other human-associated skin-resident CoNS. Closest homologs of the *hya* gene of *S. saccharolyticus* are present in two animal-associated staphylococci: *Staphylococcus agnetis* and *Staphylococcus hyicus. S. agnetis* is associated with lameness in broiler chickens, and *S. hyicus* causes skin diseases, such as exudative dermatitis in piglets [27,28]. It is tempting to suggest that the (horizontal) acquisition of *hya*, possibly from an animal-associated staphylococcal species, contributed to a lifestyle switch of *S. saccharolyticus.* Hyaluronate lyases degrade hyaluronic acid, a major polysaccharide of the extracellular matrix of tissues [29]. In the epidermis, hyaluronic acid is found in high concentrations, in particular in deeper layers of the epidermis, such as the stratum spinosum [30]. Thus, a hyaluronidase-producing *S. saccharolyticus* is likely better equipped to penetrate and propagate in deeper layers of the epidermis. We further speculate that a strong host association in deeper layers might have been established, where *S. saccharolyticus* would have access to a range of host-derived compounds including amino acids and cofactors. This in turn would render bacterial genes to synthesize such compounds dispensable. As a consequence, genome decay would be accelerated, aiming at a slimmer, less energy-consuming lifestyle that is adapted to an oxygen-depleted niche, i.e., the epidermis below the stratum corneum. 

Another open question remains regarding the clinical significance of these findings. At present, only few studies, mainly case reports, have reported the involvement of *S. saccharolyticus* in human disease. The microorganism has been described as the etiologic agent of infective endocarditis, empyema and bone and joint infections such as shoulder synovitis and vertebral osteomyelitis [31,32,33,34,35,36]. In addition, the bacterium was reported to be responsible for nosocomial bloodstream infections in a German hospital [16]. A few reports have found *S. saccharolyticus* in implant-associated infections, such as prosthetic valve endocarditis [37] and we recently described eight cases of prosthetic joint infections where *S. saccharolyticus* was identified from tissue biopsies [15]. If *S. saccharolyticus* is widespread on human skin, as our study results suggest, one would expect to see more reports regarding the potential disease association of this bacterium, as seen for example for other skin-resident CoNS, such as *S. epidermidis* and *S. capitis*. As outlined above in detail, we hypothesize that mainly due to the fastidious growth conditions, *S. saccharolyticus* was overlooked in numerous disease cases, in particular in implant-associated infections, as such infections are often caused by skin-derived bacteria, including CoNS. In several cases, *S. saccharolyticus* has been identified, but was labeled as contaminant [38]. As also true for other CoNS, assigning an etiological role to *S. saccharolyticus* in disease requires thorough investigations to exclude skin-derived contamination of the biopsy material or contamination during subsequent sample processing steps. Carefully designed and executed future studies are needed to elucidate the etiology and frequency of *S. saccharolyticus* in human disease.

The study has some limitations, most importantly the small sample size (*n* = 19) and the focus on back skin. Moreover, only relatively young participants were investigated in this study. Appropriate skin sampling methods have previously been discussed [39]; due to the here applied sampling method, i.e., skin swabs, we harvested mainly the microbiome of the stratum corneum. Thus, microorganisms that potentially penetrate deeper skin layers of the epidermis might be underrepresented. In future studies, we aim at analyzing more individuals, thereby covering and comparing different age groups and diverse skin health conditions. In addition, different skin sites will be investigated, and different skin sampling methods applied, in order to determine the specific skin tissue location of *S. saccharolyticus*.

In conclusion, here we found that the coagulase-negative species *S. saccharolyticus* is relatively often found in human skin samples, as judged from a culture-independent amplicon sequencing approach. When present, the organism can comprise a major part of the staphylococcal skin population, and is found in several different skin sites. It has yet to be shown in the future if skin that is primarily colonized with *S. saccharolyticus* has distinguishable features from skin colonized with *S. epidermidis* or *S. capitis*. 

## Supplementary Materials

The following are available online at https://www.mdpi.com/2076-2607/8/8/1105/s1, Table S1: Sequence read count matching the *tuf* gene fragment of different staphylococcal species; Table S2: Identification of sequencing reads in back skin metagenomic data that map to the *hya* gene of *S. saccharolyticus* with 100% nucleotide identity; Table S3: List of metagenomic datasets used for read mapping.
